# Maize and millet transcription factors annotated using comparative genomic and transcriptomic data

**DOI:** 10.1186/1471-2164-15-818

**Published:** 2014-09-27

**Authors:** Jinn-Jy Lin, Chun-Ping Yu, Yao-Ming Chang, Sean Chun-Chang Chen, Wen-Hsiung Li

**Affiliations:** Bioinformatics Program, Taiwan International Graduate Program, Institute of Information Science, Academia Sinica, Taipei, 115 Taiwan; Institute of Molecular and Cellular Biology, National Tsing Hua University, Hsinchu, 300 Taiwan; Biodiversity Research Center, Academia Sinica, Taipei, 115 Taiwan; Biotechnology Center, National Chung-Hsing University, Taichung, 40227 Taiwan; Department of Ecology and Evolution, University of Chicago, Chicago, IL 60637 USA

**Keywords:** Transcription factor annotation, Coregulators, Comparative genomics, Functional annotation

## Abstract

**Background:**

Transcription factors (TFs) contain DNA-binding domains (DBDs) and regulate gene expression by binding to specific DNA sequences. In addition, there are proteins, called transcription coregulators (TCs), which lack DBDs but can alter gene expression through interaction with TFs or RNA Polymerase II. Therefore, it is interesting to identify and classify the TFs and TCs in a genome. In this study, maize (*Zea mays*) and foxtail millet (*Setaria italica*), two important species for the study of C4 photosynthesis and kranz anatomy, were selected.

**Result:**

We conducted a comprehensive genome-wide annotation of TFs and TCs in maize B73 and in two strains of foxtail millet, Zhang gu and Yugu1, and classified them into families. To gain additional support for our predictions, we searched for their homologous genes in Arabidopsis or rice and studied their gene expression level using RNA-seq and microarray data. We identified many new TF and TC families in these two species, and described some evolutionary and functional aspects of the 9 new maize TF families. Moreover, we detected many pseudogenes and transposable elements in current databases. In addition, we examined tissue expression preferences of TF and TC families and identified tissue/condition-specific TFs and TCs in maize and millet. Finally, we identified potential C4-related TF and TC genes in maize and millet.

**Conclusions:**

Our results significantly expand current TF and TC annotations in maize and millet. We provided supporting evidence for our annotation from genomic and gene expression data and identified TF and TC genes with tissue preference in expression. Our study may facilitate the study of regulation of gene expression, tissue morphogenesis, and C4 photosynthesis in maize and millet. The data we generated in this study are available at http://sites.google.com/site/jjlmmtf.

**Electronic supplementary material:**

The online version of this article (doi:10.1186/1471-2164-15-818) contains supplementary material, which is available to authorized users.

## Background

Gene regulation by transcription factors (TFs) is crucial for development, maintenance of normal physiology and response to external or internal stimuli. Hence, identification and classification of TFs will increase our understanding of TF functions and regulation of biological processes. A TF contains one or more DNA-binding domains (DBDs), which bind specific DNA sequences to mediate the binding of RNA polymerase at the onset of transcription initiation. For example, Arabidopsis ethylene response factor 1 (ERF1) contains an AP2 DBD, which binds the GCC box in the promoter sequences of ethylene responsive genes , while Arabidopsis AINTEGUMENTA (ANT) contains two AP2 domains [1, 2]. A TF may also contain an auxiliary domain that facilitates DNA binding. For example, an auxin response factor (ARF) contains a B3 DBD and an auxiliary domain, the “auxin/indole-3-acetic acid (Aux/IAA) domain”. ARF proteins can form homodimers, where the two combined B3 domains can bind a TGTCTC-containing auxin responsive element, through interaction with the two Aux/IAA domains [3, 4]. In addition, there are proteins that have no DBD but can bind TFs or RNA polymerase II to alter gene regulation; such proteins are called transcription coregulators (TCs), which include coactivators and corepressors [5]. For example, an Aux/IAA protein, which contains an Aux/IAA domain, can form a heterodimer with an ARF protein and prevent the ARF from activating its target genes [4, 6, 7].

In recent years, many plant TF databases have been developed. According to Mitsuda *et al.*, in Arabidopsis approximately 70 families of TFs have been classified in public TF databases, including RARTF, AGRIS, DATF and PlnTFDB 3.0 [8–12]. Furthermore, there are other databases, such as PlantTFDB 3.0, ProFITS, GrassTFDB in Grassius, PlantTFcat and TreeTFDB, which provide classifications of TFs in Arabidopsis and other plants [13–17]. However, these databases classify TFs into families according to their own criteria, leading to differences in the number of annotated TF genes and in the number of families among databases. Moreover, only a few databases provide annotation of TCs for plants, such as ProFITS, PlnTFDB 3.0, PlantTFcat and GrassCoregDB in Grassius [11, 13–15].

We are interested in annotating TF genes and TC genes in maize and millet. Maize gives a high crop yield and is efficient in water usage. Its genome was sequenced and annotated in 2009 [18, 19]. Maize gene annotation contains ~110,000 genes in the maize Working Gene Set (WGS, release 5b) and 39,656 genes in the maize Filtered Gene Set (FGS, release 5b), in which transposons, pseudogenes, contaminants, and other low-confidence genes have been excluded. PlantTFDB 3.0, Grassius and iTAK (http://bioinfo.bti.cornell.edu/cgi-bin/itak/index.cgi) provide maize TF annotation only for the FGS genes, while most other databases have not been updated recently [13, 17]. According to the maize transcriptomes of Liu *et al.*, there were 6355 expressed genes in WGS that were not included in FGS, suggesting that some TFs and TCs have not been included in the above databases [20].

Millet is an emerging C4 model plant. It has a shorter generation time (~12 weeks vs. ~16 weeks) and a much smaller genome size (~490 Mb vs. 2500 Mb), suggesting much fewer duplicate genes and less functional redundancy compared to maize. Two different millet cultivars, Yugu1 and Zhang gu, were sequenced in 2012 [21, 22]. For millet, only PlantTFDB 3.0 provides TF annotation [17].

In this study, we conducted a comprehensive genome-wide annotation of TFs and TCs for maize WGS and also for the two millet strains, Zhang gu and Yugu1. We used protein domains to identify TFs and TCs and classified them into families in maize and millet, separately. We also identified tissue- or condition-specific TF and TC genes in maize and millet. Our study provides a database of annotated TF and TC genes in maize and millet with various kinds of supporting evidence, especially genomic and transcriptomic data. Our study sheds light on the role of different TF and TC genes in the development of different tissues in these two C4 plants.

## Results

### Genome-wide prediction and classification of TFs and TCs

To identify TF and TC genes in the maize and millet genomes, we collected all protein sequences annotated in the maize and millet genomes to form an initial set of protein sequences. Then, for each sequence we checked the presence of DBDs or TC domains.

**Figure 1:**
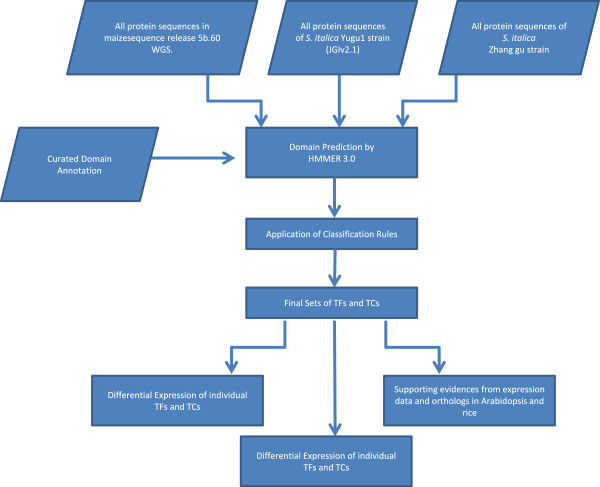
**Pipeline for TF and TC prediction and classification.**

There is abundant information of protein domains related to TFs and TCs in TF databases. To select domains that may be related to a TF or TC family, we compiled a set of related signature domains from PlantTFDB 3.0, PlnTFDB 3.0, Grassius, ProFITS and AnimalTFDB [11, 13, 14, 17, 23]. Besides TF databases, we also used other resources to find out more possible DBDs or characteristic domains of TCs. For example, Gene Ontology (GO) annotation can be used to select protein domains that may have TC function but have not been included in the TF databases we used [24]. On the other hand, experimental data such as ChIP-seq and protein binding microarray (PBM) are also useful for finding more DBDs [25, 26]. From these sources, we defined 67 TF families and 29 TC families (Additional file 1: Table S1 and Additional file 2: Table S2). As described in Methods, the domains we selected were represented by Hidden Markov Model (HMM). We used HMMER 3.0 to predict protein domains on a protein sequence. After we obtained the domain compositions on the protein sequences under study, a set of classification rules was applied to identify TFs and TCs (Methods). Figure 1 depicts the workflow of our pipeline.

By applying our pipeline, 2538 genes (3637 proteins) in maize were predicted as TF genes and classified into 64 families, including 153 genes that are not in FGS (Table 1 and Additional file 3: Table S3 (A)). In addition, 149 genes (236 proteins) were predicted as TC genes and classified into 21 families, including 8 genes that were not in FGS (Table 2 and Additional file 3: Table S3 (B)).

**Table 1:** **Numbers of TF genes in maize and millet annotated in this study**

Family ^*^	Millet Zhang gu	Millet Yugu1	Maize
**AP2**	23	24	26
**ARF**	24	24	36
**ARR-B**	9	9	9
**B3**	56	55	59
**BBR-BPC**	3	3	4
**BES1**	10	9	10
**bHLH**	177	172	207
**bZIP**	92	83	132
**C2H2**	103	108	137
**C3H**	40	38	64
**CAMTA**	7	7	8
**CO-like**	6	10	17
**CPP**	7	10	13
**CSD (N)**	2	0	4
**DBB**	8	8	14
**Dof**	24	28	46
**E2F/DP**	8	7	20
**EIL**	8	7	9
**ERF**	126	143	205
**FAR1**	8	61	19
**G2-like**	51	44	66
**GATA**	30	28	43
**GeBP**	12	15	21
**GRAS**	62	57	101
**GRF**	7	10	15
**HB-other**	9	8	28
**HB-PHD**	2	2	4
**HD-ZIP**	49	46	65
**HMG (N)**	10	10	15
**HMGI/HMGY (N)**	6	6	13
**HRT-like**	1	1	0
**HSF**	25	22	29
**LBD**	32	32	44
**LFY**	1	1	2
**LSD**	5	5	6
**MBD (N)**	12	12	13
**MIKC**	31	22	43
**mTERF (N)**	53	55	31
**M-type**	40	42	44
**MYB**	128	121	169
**MYB_related**	80	76	143
**NAC**	139	141	139
**NF-X1**	2	2	4
**NF-YA**	9	10	18
**NF-YB**	16	15	19
**NF-YC**	16	14	19
**Nin-like**	18	17	18
**Pseudo ARR-B (N)**	5	4	5
**RAV**	2	4	3
**S1Fa-like**	1	1	2
**SBP**	20	18	38
**SRS**	6	6	13
**STAT**	1	1	1
**TALE**	24	24	29
**TCP**	17	18	46
**Trihelix**	27	27	48
**VOZ**	2	2	6
**Whirly**	2	2	2
**WOX**	9	13	20
**WRKY**	110	109	128
**YABBY**	8	8	13
**BED (N)**	9	13	54
**ZF-HD**	10	8	22
**LITAF (N)**	1	1	1
**MIZ (N)**	5	3	2

*: N at the end of a family name means that the family is not defined in PlantTFDB3.0.

**Table 2:** **Numbers of TC genes in maize and millet annotated in this study**

Family	Millet Zhang gu	Millet Yugu1	Maize
**AUX/IAA**	29	29	52
**GIF**	3	3	3
**MBF1**	2	2	3
**Med10**	1	1	2
**Med11**	1	1	2
**Med12**	1	1	6
**Med13_C**	1	1	1
**Med14**	2	2	1
**Med17**	1	1	2
**Med18**	1	1	0
**Med20**	1	1	1
**Med22**	1	1	1
**Med31**	1	1	1
**Med4**	1	1	2
**Med6**	1	1	2
**Med7**	2	2	2
**PC4**	2	2	3
**RB**	2	2	5
**Sigma54_activat**	9	10	9
**Spt20**	2	2	1
**TAZ**	7	7	7
**OFP**	33	27	43

In millet Yugu1, 1880 genes (2116 proteins) were predicted as TF genes and classified into 64 families (Table 1 and Additional file 3: Table S3 (C)). In addition, 99 genes (118 proteins) were predicted as TC genes and classified into 22 families (Table 2 and Additional file 3: Table S3 (D)).

In millet Zhang gu, 1846 genes were predicted as TF genes and classified into 65 families (Table 1 and Additional file 3: Table S3 (E)), while 104 genes were predicted as TC genes and classified into 22 families (Table 2 and Additional file 3: Table S3 (F)). Orthologs of maize and millet TF and TC genes in other genomes

To gain additional support of identified TF and TC genes, we checked the existence of orthologous genes in other plant species. For a maize TF or TC gene predicted by our pipeline, we examined whether it has orthologs in *Arabidopsis thaliana* or rice (*Oryza sativa japonica*) (Ensembl Plants release 17) because the Arabidopsis and rice genomes are well annotated [27]. We found 2392 predicted maize TF genes (94.25%) have orthologs in either or both of the two reference species, including 107 genes that were not included in maize FGS (Table 3 and Additional file 3: Table S3 (A)). The corresponding number for TC genes was 143 (95.97%) (Table 4 and Additional file 3: Table S3 (B)), including 7 genes that were not included in maize FGS. Thus, a substantial proportion of maize TF and TC genes not included in FGS have supporting evidence in Arabidopsis or rice.

**Table 3:** **Numbers of TF genes in maize and millet with orthologs in other species and support from expression data**

	Maize	Millet Yugu1	Millet Zhang gu
**Number of TF genes**	2538	1880	1846
**TF genes with orthologs in Arabidopsis or rice**	2392	1808	1680
**TF genes regarded as expressed**	2341	N/A	1397
**TF gene with orthologs in maize**	N/A	1537	1701
**TF gene with orthologs in millet Yugu1**	2209	N/A	1772
**TF gene with orthologs in millet Zhang gu**	2249	1799	N/A

**Table 4:** **Numbers of TC genes in maize and millets with orthologs in other species and support from expression data**

	Maize	Millet Yugu1	Millet Zhang gu
**Number of TC genes**	149	99	104
**TC genes with orthologs in Arabidopsis or rice**	143	97	95
**TC genes regarded as expressed**	145	N/A	91
**TC gene with orthologs in maize**	N/A	90	98
**TC gene with orthologs in millet Yugu1**	136	N/A	99
**TC gene with orthologs in millet Zhang gu**	132	97	N/A

In millet Yugu1, 1808 TF genes (96.17%) and 97 TC genes (97.97%) have orthologs in Arabidopsis or rice (Tables 3 and 4, Additional file 3: Table S3 (C) and S3 (D)). In millet Zhang gu, the orthologous relationships were not covered in Ensembl Plants, so we used BLASTP to search for putative orthologs of TFs and TCs of millet Zhang gu in Arabidopsis and rice (Methods) [27]. We found 1680 TF genes (91.01%) and 95 TC genes (91.35%) have orthologs in Arabidopsis or rice (Tables 3 and 4, Additional file 3: Table S3 (E) and S3 (F)).

We also examined orthology between maize and millet because we also need to consider the possibility that some TFs and TCs appeared after panicoideae emerged. Between maize and millet Yugu1, we found 2209 maize TF genes (87.04%) and 136 TC genes (91.28%) have orthologs in millet Yugu1 (Tables 3 and 4, Additional file 3: Table S3 (A) and S3 (B)), while 1537 millet Yugu1 TF genes (81.76%) and 90 TC genes (90.91%) have orthologs in maize (Tables 3 and 4, Additional file 3: Table S3 (C) and S3 (D)). Between maize and millet Zhang gu, 2249 maize TF genes (88.61%) and 132 maize TC genes (91.03%) have orthologs in millet Zhang gu (Tables 3 and 4, Additional file 3: Table S3 (A) and S3 (B)), while 1701 millet Zhang gu TF genes (92.15%) and 98 TC genes (94.23%) have orthologs in maize (Tables 3 and 4, Additional file 3: Table S3 (E) and S3 (F)).

In conclusion, a very high proportion of the TF and TC genes we predicted in maize and millet have orthologs in other species.

### Expression of maize TF and TC genes in different tissues or conditions

To gain functional support of our annotated TF and TC genes, we collected the FPKM (Fragments Per Kilobase of exon per Million fragments mapped) values of maize genes from 7 RNA-seq datasets via qTeller (http://qteller.com), time course transcriptomes from Liu *et al.*, and a microarray dataset from Sekhon *et al.* to examine their expression in different tissues or under different conditions [20, 28–35]. We found that in maize, 2287 TF genes (90.11%) and 143 TC genes (98.11%) were expressed in at least one RNA-seq dataset (Additional file 3: Table S3 (A) and S3 (B), Additional file 4: Table S4 and Additional file 5: Table S5), and 1838 TF genes (72.42%) and 120 TC genes (80.54%) were expressed in at least one condition in the microarray dataset (Additional file 3: Table S3 (A) and S3 (B)). When all 9 datasets were considered together, 2341 TF genes (92.24%) and 145 TC (97.32%) genes were found to be expressed (Tables 3 and 4, Additional file 3: Table S3 (A) and S3 (B)).

**Figure 2:**
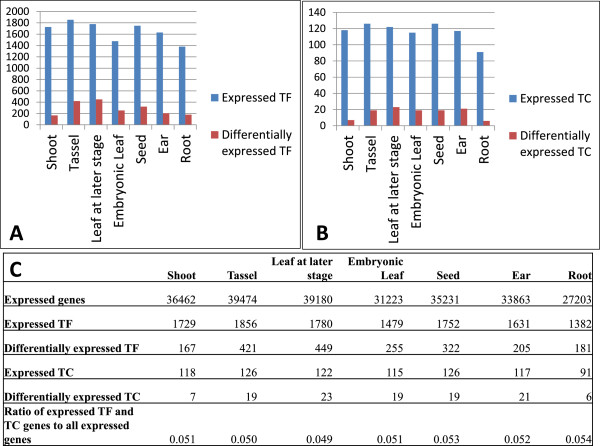
**Expression of maize TF and TC genes in different tissues.** Number of expressed or preferentially expressed TF **(A)** and TC **(B)** genes in different tissues is shown. Complete statistics is shown in **(C)**.

To identify tissue expression preference of TF and TC genes, we divided the RNA-seq datasets of maize into 7 different tissue groups. The ratios of expressed TF and TC genes to all expressed genes are around 5% in different tissues (Figure 2), comparable to the ratio in Arabidopsis [10].

**Figure 3:**
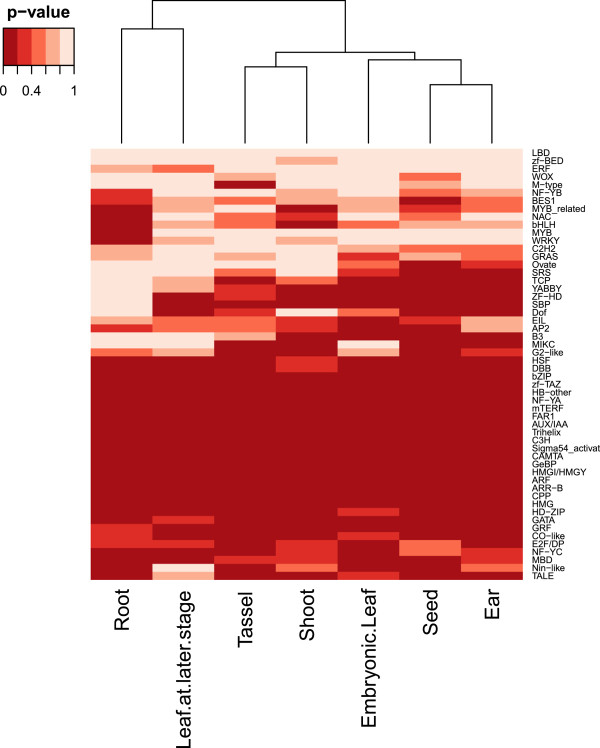
**Clustering of enrichment pattern of expressed TF and TC genes in different tissues in maize.** Each cell in the figure indicates the significance level (in terms of p-value) of a TF family in a tissue group as indicates in Table 5. The dendrogram depicted the similarity of enrichment pattern among different tissue groups.

We studied the expression enrichment of TF and TC families in different tissues. We found that 39 TF families and 4 TC families were enriched in at least one tissue group (Table 5). Interestingly, there are 9 families (AUX/IAA, C3H, CAMTA, FAR1, GeBP, NF-YA, Sigma54_activat, Trihelix and mTERF) that are enriched in all tissue groups, implying that these families may play regulatory roles in all tissues. For example, the WRKY family was only enriched in root, suggesting that WRKY genes are important in maize root, as in Arabidopsis root [36]. The MIKC family, which possesses a MADS domain, is enriched in floral organs and seeds, so they may be important in floral organ and seed development, as in Arabidopsis [37]. OFP and B3 families are enriched in the seed. As some OFP genes affect fruit development in pepper and B3 genes affect seed maturation and embryo development in Arabidopsis, they may play important roles in seed development in maize [38–42]. The YABBY family is important in determining the abaxial cell fate in lateral organs in Arabidopsis and lateral organ outgrowth in maize, and it is highly enriched in the maize ear and embryonic leaf data we collected [43, 44]. The G2-like family is enriched in shoot. The KANADI genes in the G2-like family are known to be involved in the development of organ polarity in Arabidopsis [45]. Our data suggests that members in the G2-like family play various roles in shoot development.We also studied whether similar tissues would show similar enrichments of TF and TC families (Figure 3). Indeed, some closely related tissues showed highly similar enrichments of TF and TC families. For example, the enrichment patterns in embryonic leaf, seed and ear are more similar than that among other tissues. The two inflorescence types in maize are derived from the tip of shoot, and our result showed that their TF and TC enrichment patterns are similar. Thus, tissues similar in function tend to express similar TF and TC genes.

**Table 5:** **Significance levels of TF and TC Families in different maize tissues**

Family	Ear	Embryonic Leaf	Leaf at later stage	Root	Seed	Shoot	Tassel
**AP2**	0.67969	0.07021	0.56658	0.34981	0.03766	0.33074	0.59394
**ARF**	4.86E-05	3.88E-05	0.06251	0.00087	0.00118	0.003	0.00512
**ARR-B**	0.05005	0.02615	0	0.06493	0.06871	0.09044	0
**AUX/IAA**	0.01157	0.00349	0.01556	0.00023	0.00152	0.00134	0.0014
**B3**	0.07468	0.05294	0.974	0.88084	0.04704	0.09984	0.78141
**BES1**	0.43059	0.56894	0.72694	0.15304	0.04916	0.57422	0.44143
**C2H2**	0.40462	0.78835	0.93476	0.73105	0.48738	0.97906	0.97111
**C3H**	4.24E-06	5.48E-07	0.00079	9.57E-07	4.66E-08	3.25E-07	0.00022
**CAMTA**	0	0	0	0	0	0	0
**CO-like**	0.0025	0.24574	0	0.3737	0.03475	0	0
**CPP**	0.0112	0.00423	0	0.13826	0	0.0272	0
**DBB**	0	0.02279	0.05128	0	0.01288	0.30478	0.05652
**Dof**	0.03949	0.43131	0.17721	0.92732	0.01726	0.85531	0.33311
**E2F/DP**	0.17299	0.00468	0.2946	0.29741	0.44409	0.32046	0.12153
**EIL**	0.78314	0.02615	0.49092	0.7444	0.28728	0.34398	0.5084
**ERF**	1	0.98559	0.54626	0.79527	0.90577	0.99998	0.99987
**FAR1**	0.01502	0.00468	0.02056	0.00061	0.00338	0	0
**G2-like**	0.30988	0.77284	0.61504	0.47687	0.18978	0.04774	0.1884
**GATA**	0.01486	0.00012	0.22716	0.19129	0.00329	0.02585	0.05481
**GRAS**	0.48999	0.37522	0.92927	0.60385	0.60767	0.98753	0.70509
**GRF**	0	0	0.04081	0.34429	0	0	0
**GeBP**	0	0	0	0.00524	0	0	0
**HB-other**	0.11041	0.10066	0.12306	0.05348	0.17347	0.11616	0.13742
**HD-ZIP**	0.00013	0.20318	0.06448	0.03399	0.00064	0.03699	0.00026
**HMG**	0.00529	0.1858	0.04081	0.01778	0.06054	0.08808	0.04531
**HMGI/HMGY**	0	0.00423	0.06445	0.00081	0	0	0.0705
**HSF**	0.01116	4.94E-05	0.0551	0.02572	0.0236	0.22818	0.16553
**LBD**	0.99964	0.99862	0.99999	0.97509	0.92321	0.99993	0.99971
**M-type**	0.85675	0.9992	0.96814	0.92997	0.6328	0.92136	0.16782
**MBD**	0.22458	0.00423	0.06445	0.13826	0.10394	0.36196	0.27978
**MIKC**	0.00118	0.99996	0.99983	0.94689	0.01181	0.12968	0.00129
**MYB**	0.99839	1	0.99999	0.04121	0.84955	0.97306	0.97846
**MYB_related**	0.56902	0.77282	0.71872	0.00482	0.23988	0.13373	0.97806
**NAC**	0.99593	0.99509	0.93794	0.07169	0.49051	0.21413	0.41416
**NF-YA**	0.00364	0.00108	0	0.04416	0.0066	0.01104	0.03632
**NF-YB**	0.61894	0.80516	0.91116	0.20323	0.50517	0.78466	0.80344
**NF-YC**	0.34727	0.19528	0.02056	0.15186	0.44409	0.32046	0.02334
**Nin-like**	0.47377	0.14231	0.82469	0.12431	0.15546	0.42769	0.03632
**OFP**	0.32285	0.45214	0.86382	1	0.02237	0.83652	0.9731
**SBP**	0.00085	0.08131	0.03956	0.83484	0.00254	0.0064	0.00028
**SRS**	0.0112	0.30507	0.99933	0.96581	0	0.98247	0.56448
**Sigma54_activat**	0	0	0	0	0	0	0
**TALE**	0.00038	0.3918	0.65757	0.00274	0.00103	0.0024	0.01626
**TCP**	0.10247	0.06195	0.7239	0.99872	0.09356	0.42335	0.02011
**Trihelix**	2.10E-05	3.09E-09	0.00386	3.27E-06	5.58E-07	0.00161	0
**WOX**	0.94866	0.98276	0.99999	0.97133	0.44409	0.99912	0.76411
**WRKY**	0.99002	0.99987	0.68042	0.00066	0.99999	0.76124	0.97375
**YABBY**	0.0112	0.0335	0.7832	0.9925	0.10394	0.14156	0.27978
**ZF-HD**	0.00056	0.09192	0.06354	1	0	0.01969	0.21141
**bHLH**	0.76136	0.40417	0.64008	0.06933	0.61467	0.10359	0.50928
**bZIP**	0.0256	0.03162	0.01097	8.05E-08	2.45E-05	0.10417	0.12073
**mTERF**	0.00076	7.17E-06	0.00263	0.0492	0.00188	0.00041	0.02373
**BED**	0.86112	0.99946	0.99932	0.99858	0.99998	0.75519	0.80988
**TAZ**	0.10583	0	0	0	0	0.16493	0

Significance levels are based on P-values reported by Fisher’s exact test, a lower significance level means that larger amount of genes in a family are expressed in a tissue group. A P-value lower than 0.05 is regarded as significant.

### Expression of millet TF and TC genes in different tissues or conditions

**Figure 4:**
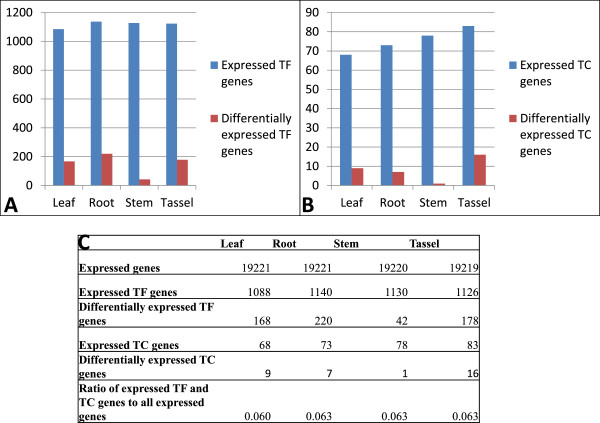
**Expression of millet TF and TC genes in different tissues.** Number of expressed or preferentially expressed TF **(A)** and TC **(B)** genes in different tissues is shown. Complete statistics is shown in **(C)**.

For millet Zhang gu, RNA-seq samples from 4 different tissues were available (Additional file 4: Table S4), and we identified **1397** TF genes (**75.68%**) and 91 TC genes (87.5%) expressed under the criterion of having the adjusted RPKM value ≥1 in at least one tissue (Tables 3 and 4, Additional file 6: Table S6). The proportion of TF and TC genes with supporting evidence from gene expression data in millet Zhang gu is lower than that in maize TF and TC genes, because gene expression data is far less abundant in millet than in maize. The ratios of expressed TF and TC genes to all expressed genes are around 6% in different tissues (Figure 4).

**Figure 5:**
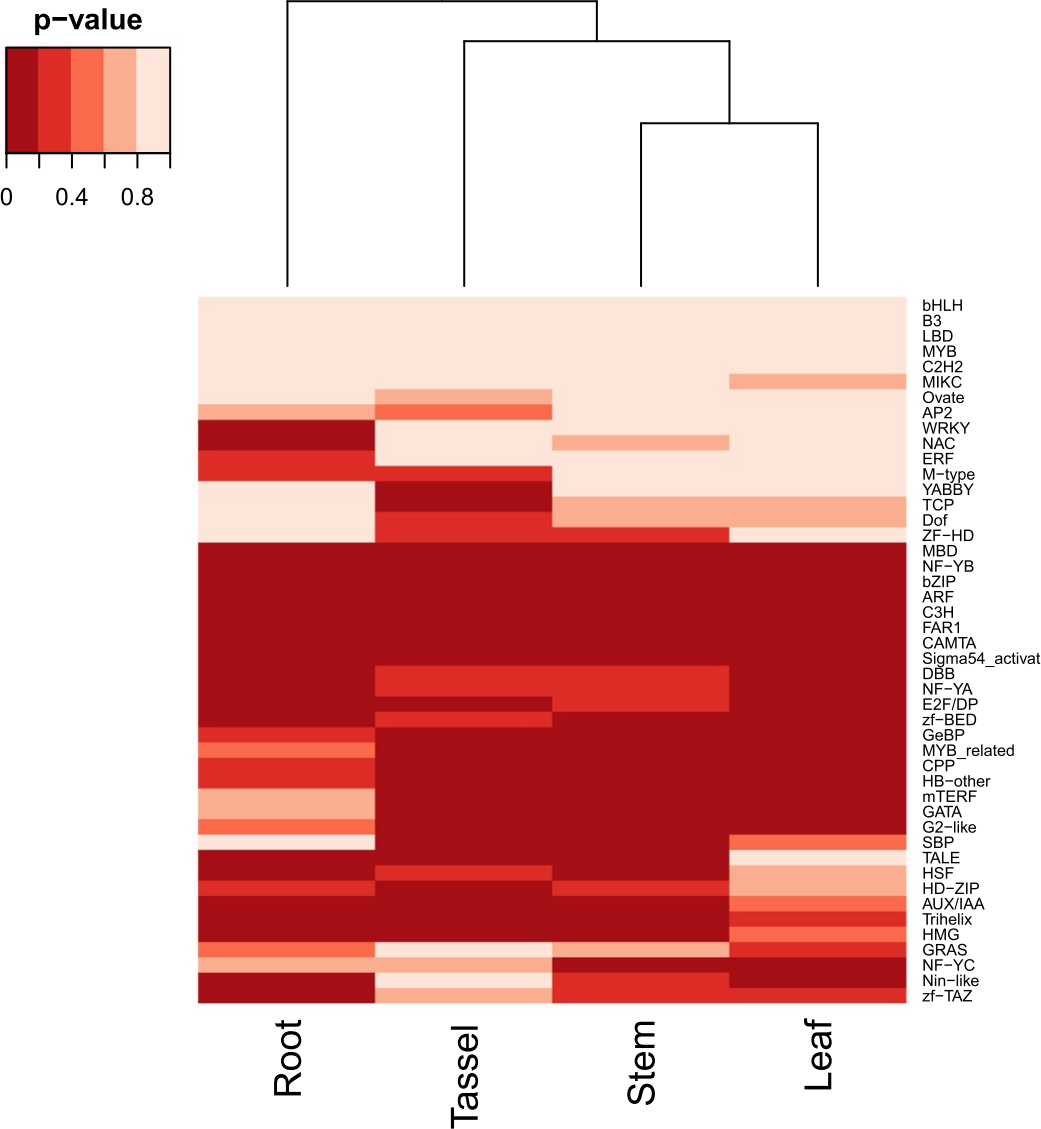
**Clustering of enrichment pattern of expressed TF and TC genes in different tissues in millet.** Each cell in the figure indicates the significance level (in terms of p-value) of a TF family in a tissue group as indicated in Table 6. The dendrogram depicted the similarity of enrichment pattern among different tissue groups.

We also conducted expression enrichment analysis in millet Zhang gu. There were 28 TF families and 2 TC families enriched in at least one tissue group (Table 6), and 3 TF families and 1 TC family enriched in all 4 tissue groups (C3H, CAMTA, FAR1 and Sigma54_activat). The enrichment patterns of TF and TC families in the four tissue groups are shown in Figure 5. These families are also enriched in all tissue groups of maize, so they may be important in regulating biological processes. The expression of the mTERF family is not significantly enriched in root (Fisher’s exact test p-value = 0.76). In Arabidopsis, the number of mTERF genes potentially expressed in root tissues is lower than in other tissues, and the enrichment test in maize was barely significant (Fisher’s exact test p-value = 0.049) [46].

**Table 6:** **Significance levels of TF and TC Families in different millet tissues**

Family	Leaf	Root	Stem	Tassel
**AP2**	0.99945	0.76718	0.98899	0.56275
**ARF**	0.01159	0.06865	0.01158	0
**AUX/IAA**	0.47105	0.03618	0.03614	0.00532
**B3**	0.99396	0.95681	0.99811	0.95669
**C2H2**	0.89246	0.96644	0.82544	0.99639
**C3H**	0.00137	0	0.00137	0.00137
**CAMTA**	0	0	0	0
**CPP**	0	0.31277	0	0
**DBB**	0	0	0.25758	0.25749
**Dof**	0.77736	0.9161	0.77722	0.26481
**E2F/DP**	0	0	0.25758	0
**ERF**	0.81309	0.38687	0.97696	0.99993
**FAR1**	0	0	0	0
**G2-like**	0.14324	0.59204	0.00409	0.01937
**GATA**	0	0.6566	0.01406	0.08018
**GRAS**	0.21535	0.54695	0.70613	0.8295
**GeBP**	0	0.39791	0.11863	0.11857
**HB-other**	0	0.25768	0	0
**HD-ZIP**	0.64978	0.31871	0.31847	0.07824
**HMG**	0.51145	0.17489	0.17481	0
**HSF**	0.69426	0	0.09377	0.25772
**LBD**	1	0.87495	0.99906	0.99983
**M-type**	0.95506	0.34874	0.98958	0.34845
**MBD**	0.17489	0.17489	0.17481	0
**MIKC**	0.73159	0.99674	0.99908	0.93195
**MYB**	1	0.83698	0.99173	0.97335
**MYB_related**	0.02464	0.48928	0.0246	0.06101
**NAC**	0.98971	0.04414	0.79086	0.99741
**NF-YA**	0	0	0.2122	0.21211
**NF-YB**	0.17489	0.17489	0	0.17473
**NF-YC**	0	0.69635	0.11863	0.69607
**Nin-like**	0.1187	0.1187	0.39777	0.88753
**OFP**	1	1	0.99653	0.61825
**SBP**	0.40284	0.83409	0.03707	0
**Sigma54_activat**	0	0	0	0
**TALE**	0.98772	0.06865	0.06859	0.06852
**TCP**	0.73579	0.89168	0.73563	0
**Trihelix**	0.36847	0.0586	0.00954	0.05848
**WRKY**	0.96298	0.00631	0.96289	1
**YABBY**	0.89056	0.99982	0.89049	0
**ZF-HD**	0.89056	0.97861	0.25758	0.25749
**bHLH**	0.99999	0.99964	0.99472	0.95644
**bZIP**	0.07649	0.01792	0.07635	0.01784
**mTERF**	0.00206	0.76309	0.00205	0.00205
**BED**	0	0	0	0.31257
**TAZ**	0.31277	0	0.31267	0.71374

Significance levels are based on P-values reported by Fisher’s exact test, a lower significance level means that larger amount of genes in a family are expressed in a tissue group. A P-value lower than 0.05 is regarded as significant.

### TF and TC families enriched in same tissues in millet and maize

We cross-compared our result of enrichment analysis in maize and millet and identified 18 TF families and 3 TC families enriched significantly in at least one same tissue of maize and millet (Table 5, Table 6, Figure 3 and Figure 5). Besides the four families that were enriched in all tissue groups in maize and millet, other TF and TC families may also play important roles in tissue groups they enriched. For example, the WRKY family was enriched in both maize and millet roots, implying the importance of WRKY genes in the root. The two families NF-YA and NF-YC, which are related to the nuclear factor Y complexes, were enriched in leaf [47]. A previous study in rice indicated that their possible binding DNA motifs were enriched in coexpressed genes in specific leaf cell types in rice, so it is possible that these two families also play an important role in leaf development in grasses [48]. The SBP family was enriched in the tassel group. A previous study indicated that it is enriched for proximity to tassel branching loci, so this family may be important in tassel development [49]. The NAC family was significantly enriched in millet root and although the enrichment of the NAC family in maize root was not significant (p-value = 0.072), the number of NAC family genes expressed in root was much larger than in the other tissues studied. Previous studies found some NAC family genes expressed in different zones and at developmental stages of the root in Arabidopsis and poplar, suggesting that NAC genes are important for plant root development [50–54].

### TF and TC genes preferentially expressed among tissues

We examined TF and TC genes preferentially expressed among tissues in maize or millet. In maize, we identified 1819 TF genes and 109 TC genes preferentially expressed in at least one tissue according to the available RNA-seq data (Additional file 3: Table S3 (A) and S3 (B)). In millet, we identified 608 TF genes and 33 TC genes preferentially expressed in at least one tissue (Additional file 3: Table S3 (E) and S3 (F)). Moreover, some TFs and TCs were preferentially expressed in a set of physiologically similar tissues.

In addition, we identified 138 TF and 7 TC genes preferentially expressed in the same types of tissue in maize and millet (Additional file 3: Table S3 (A), S3 (B), S3 (E) and S3 (F)). For example, *GOLDEN2* (maize gene ID: GRMZM2G087804, millet Zhang gu gene ID: Millet_GLEAN_10019358) and *GLK1* (maize gene ID: GRMZM2G026833, millet Zhang gu gene ID: Millet_GLEAN_10029030), both of which are involved in chloroplast development, were both preferentially expressed in maize and millet leaves (Additional file 3: Table S3 (A) and S3 (E)) [55, 56]. *APETALA3* (maize gene ID: GRMZM2G139073, millet Zhang gu gene ID: Millet_GLEAN_10022237) and *PISTILLATA* (maize gene ID: GRMZM2G110153, millet Zhang gu gene ID: Millet_GLEAN_10010374), which are important for the specification of floral organs in Arabidopsis, were preferentially expressed in tassel in maize and in millet (Additional file 3: Table S3 (A) and S3 (E)) [57]. *FIT1* (maize gene id: GRMZM2G173521, millet Zhang gu gene id: Millet_GLEAN_10021794), which is important in iron deficiency response in Arabidopsis root, was preferentially expressed in root in maize and in millet (Additional file 3: Table S3 (A) and S3 (E)) [58]. Conservation in tissue preference of gene expression may imply conservation in function.

### Possible C4-related TF and TC genes in maize and millet

As we are annotating TF and TC genes in maize and millet, which are C4 plants, it is interesting to know whether some of the TF and TC genes we identified are related to C4 photosynthesis or kranz anatomy development. Here, we considered C4-related TF and TC genes in two different aspects: (a) their possible contributions to regulatory differences between bundle sheath and mesophyll cells, and (b) their possible contributions to the formation of kranz anatomy. In maize, we annotated 995 TF genes and 71 TC genes possibly related to C4 characteristics (Additional file 7: Tables S7 (A) and S7 (B)). In millet, the corresponding numbers are 546 TF genes and 33 TC genes (Additional file 7: Tables S7 (C) and S7 (D)).

C4 photosynthesis is coordinated between bundle sheath (BS) and mesophyll (M) cells. Thus, it is interesting to study the regulatory differences between BS and M cells. For this purpose, Li *et al*. and Chang *et al*. obtained BS and M cell transcriptomes [29, 34]. Here we used the data of Chang *et al*. because it was more abundant. We identified 256 TF genes and 16 TC genes preferentially expressed in BS cells; among which 44 TF genes and 14 TC genes were not identified by Chang *et al*. (Additional file 7: Tables S7 (A) and S7 (B)). We identified 64 TF genes and 3 TC genes preferentially expressed in M cells, among which 11 TF genes and 3 TC genes were not identified by Chang *et al*. (Additional file 7: Tables S7 (A) and S7 (B)).

John *et al*. identified genes differentially expressed between BS and M cells in green foxtail (*Setaria viridis*) and conducted a detailed comparison of the gene expression patterns in BS and M cells in green foxtail and maize [59]. Using their data, we identified 446 TF genes and 32 TC genes preferentially expressed in BS cells (Additional file 7: Tables S7 (A) and S7 (B)), among which 133 TF genes and 10 TC genes have conserved BS-preference in their syntenic orthologs in millet (Additional file 7: Tables S7 (A) and S7 (B)). We identified 303 TF genes and 24 TC genes preferentially expressed in M cells; among which 74 TF genes and 7 TC genes have conserved M-preference in their syntenic orthologs in millet (Additional file 7: Tables S7 (A) and S7 (B)). We identified 35 TF genes and 9 TC genes preferentially expressed in BS cells that were not previously annotated as TF genes or TC genes, among which 11 TF genes and 5 TC genes have conserved BS-preference in their syntenic orthologs in millet (Additional file 7: Tables S7 (A) and S7 (B)). We identified 16 TF genes and 10 TC genes preferentially expressed in M cell that were not annotated as TF genes or TC genes in the original data, among which 1 TF gene and 3 TC genes have conserved M-preference in their syntenic orthologs in millet (Additional file 7: Tables S7 (A) and S7 (B)).

Tausta *et al*. isolated BS and M cells from 3 different positions of maize leaves to study the developmental dynamics of BS and M cells at different photosynthetic stages and they identified maize genes differentially expressed between BS and M cells at these stages [60]. Using their data, we identified 269 TF genes and 13 TC genes preferentially expressed in BS cells, including 25 TF genes and 5 TC genes that were not previously annotated as TF genes or TC genes (Additional file 7: Tables S7 (A) and S7 (B)). Moreover, we identified 203 TF genes and 15 TC genes preferentially expressed in M cells. Among them, 25 TF genes and 6 TC genes were not annotated as TF genes or TC genes in the original data (Additional file 7: Tables S7 (A) and S7 (B)).

The gene expression dynamics in leaves with and without kranz anatomy can also provide clues for finding possible key regulators of kranz anatomy. Wang *et al*. collected RNA samples from maize foliar (with kranz anatomy) and husk leaves (without kranz anatomy) and identified some candidate regulators that may regulate the formation of kranz anatomy [61]. Using their data, we identified 40 TF genes and 1 TC genes that may be positive regulators of kranz anatomy, 3 of which were not annotated as TF genes in the original study (Additional file 7: Tables S7 (A) and S7 (B)). We identified 29 TF genes that may be negative regulators of kranz anatomy, 7 of which were not annotated as TF genes in the original study (Additional file 7: Tables S7 (A) and S7 (B)).

In millet, John *et al*. used green foxtail (*Setaria viridis*) to identify genes differentially expressed between M and BS cells [59]. Using their data, we identified 304 TF and 17 TC genes preferentially expressed in BS cells. Among them, 133 TF genes and 10 TC genes have conserved BS-preference in their syntenic orthologs in maize (Additional file 7: Tables S7 (C) and S7 (D)). We identified 242 TF genes and 16 TC genes preferentially expressed in M cells. Among them, 75 TF genes and 8 TC genes have conserved M-preference in their syntenic orthologs in maize (Additional file 7: Tables S7 (C) and S7 (D)). Also, we identified 28 TF genes and 5 TC genes preferentially expressed in BS cells that had not previously been annotated as TF or TC genes. Among them, 9 TF genes and 4 TC genes have conserved BS-preference in their syntenic orthologs in maize (Additional file 7: Tables S7 (C) and S7 (D)). We identified 13 TF genes and 4 TC genes preferentially expressed in M cells that had not previously been annotated as TF or TC genes. Among them, 4 TF genes and 2 TC genes have conserved M-preference in their syntenic orthologs in maize (Additional file 7: Tables S7 (C) and S7 (D)).

In summary, we identified 995 TF genes and 71 TC genes in maize and 546 TF genes and 33 TC genes in millet that are potentially related to C4 photosynthesis or kranz anatomy development (Additional file 7: Tables S7 (A) ~ (D)).

## Discussion

### Annotation of maize and millet TF and TC genes

Our annotation of TFs and TCs in maize and two millet strains is based on the DBDs and TC domains considered by many plant TF databases and servers such as PlantTFDB 3.0, Grassius, PlnTFDB 3.0, ProFITS and PlantTFcat [11, 13–15, 17]. We mainly followed the TF family definition of PlantTFDB 3.0, but information from other plant TF databases, animalTFDB, Gene Ontology and experimental data such as PBM and ChIP-seq data, were also taken as source for identifying TF and TC families [23–26]. We revised the TF family classification in PlantTFDB 3.0 and defined 9 additional TF families (Additional file 1: Table S1) [17]. We predicted 2538 TF genes and 149 TC genes in maize, 1880 TF genes and 99 TC genes in millet Yugu1, and 1846 TF genes and 104 TC genes in millet Zhang gu (Table 1).

For maize, our consideration of WGS genes helped identified more TF genes. Current TF databases such as PlantTFDB 3.0, Grassius and iTAK (http://bioinfo.bti.cornell.edu/cgi-bin/itak/index.cgi) use the FGS of maize gene annotation, because genes in FGS have better experimental support than non-FGS genes [13, 17]. However, as extensive maize gene expression data are now available and can be used to support gene predictions, we used WGS. To gain additional support for our predictions, we searched for homologs in the *Arabidopsis thaliana* and rice (*Oryza sativa japonica*) genomes, which are well assembled and annotated. By considering WGS, we obtained 153 TF genes and 8 TC genes not covered by FGS (Additional file 3: Tables S3 (A) and S3 (B)). For the TF families in PlantTFDB 3.0, we obtained 108 TFs that are not in FGS, 57 of which have orthologs in rice or Arabidopsis and also have support from gene expression data, 45 of which only have one type of support, while 6 of which have neither type of support (Additional file 3: Table S3 (A)) [17]. For the two millet strains, PlantTFDB 3.0 covered only the TF annotation of Yugu1. Our study covered TF annotation of both genomes. In both genomes, at least 95% of the TF genes in our database have orthologs in rice or Arabidopsis (Table 3) [17].

The differences in the numbers of TF and TC genes between maize and millet are noticeable due to the large difference in genome size between maize and millet and in their numbers of functional genes. Indeed, the current estimates of protein-coding genes are 39,000 ~ 64,000, 35,471 and 38,801 for maize, millet Yugu1 and millet Zhang gu, respectively. Another reason is that the maize genome is more completely sequenced and better annotated than millet. In fact, the current assemblies of the two millet strains only contain about 81% ~ 86% of the genome [21, 22]. The ratios of the number of TF and TC genes to that of all genes are 4.2%, 6.9%, 5.6% and 5.0% for maize WGS, maize FGS, millet Yugu1 and millet Zhang gu, respectively, which are approximately equal to the ratio in Arabidopsis (5-10%) [10]. We identified 995 TF genes and 71 TC genes in maize that may potentially contribute to C4 characteristics in maize (Additional file 7: Tables S7 (A) ~ (D)). These TF and TC genes were preferentially expressed in BS or M cells in maize and possibly regulates cell-specific processes, or are preferentially expressed in foliar leaves or husk leaves, so that they may be possible positive or negative regulators of kranz anatomy development.

### Phylogenies and possible functions of newly annotated TF families

From plant TF databases and literature we collected 9 TF families not included in PlantTFDB 3.0; most of them have only been defined in AnimalTFDB, PlnTFDB 3.0, ProFITS, Grassius and PlantTFcat but were not regarded as DNA-binding TFs [11, 14, 15, 17, 23]. We constructed their phylogenies to infer the evolutionary relationships between TF genes in a family and their relatives in other species (Additional file 8: Figure S1-S9 and Additional file 9: Table S8). Among these 9 families, only maize FGS TFs in the mTERF family were annotated by GrassTFDB in Grassius and in another study but none of the known plant TF databases cover all of these 9 families of maize and millet genes [13, 62]. In these TF families, we have identified 138 new TF genes in maize, 104 new TF genes in millet Yugu1 and 103 new TF genes in millet Zhang gu (Table 1). The proportion of TF genes in additional families is around 5% in the whole set of TFs in either maize or millet.

For the Pseudo ARR-B family, whose family members contain a response regulator domain at the N terminus and a CCT domain at the C terminus. *In vitro* assays indicated that proteins in this family can bind to DNA via their CCT domain [25]. Many members of Pseudo ARR-B families have been known to be involved in the regulation of circadian rhythm [63]. We conducted a phylogenetic analysis of the members of this family in maize, millet, Arabidopsis, rice, spiked moss, moss and green algae (Additional file 8: Figure S1). The members of this family can be divided into three clades: *TOC1*, *PRR3*/*PRR7* and *PRR5*/*PRR9*. The *TOC1* clade is more ancient than the other 2 clades. Our phylogeny is concordant with those of previous studies [64, 65]. The mTERF family broadly exists in flowering plants but is not found in fungi [66]. Proteins in this family mainly target plastids and mitochondria. Its possible functions include termination of mitochondrial gene transcription and plastid gene expression. Our phylogenetic analysis generated a tree with the topology similar to those of the previous studies [62, 66] (Additional file 8: Figure S2).

The MBD family proteins can bind to methylated DNA. They usually act as transcriptional repressors, possibly by interacting with histone deacetylase [67]. They can bind methylated sequences, preventing them from interacting with other TFs, by altering chromatin structure and possibly by sequestering other TFs [68]. The phylogeny of this family (Additional file 8: Figure S3) is generally in agreement with that of the previous study [69].

The LITAF family derived its name from its characteristic domain resembling the lipopolysaccharide-induced tumour necrosis alpha factor (LITAF). It is much less abundant in plants. In Arabidopsis, rice, maize and millet, this family contains only one gene. In Arabidopsis, it interacts with *LESION SIMULATING DISEASE1* (*LSD1*) and negatively regulates hypersensitive cell death [70]. As some members of this family in human may be involved in programmed cell death, this function may have been conserved across divergent evolutionary lineages, including maize and millet [71, 72]. The phylogenetic tree of this family (Additional file 8: Figure S4) generally agrees with the one on TreeFam (tree id: TF313294) but with small discrepancies with primate LITAF members [73]. The BED family members have a BED-type zinc finger. This domain appears in many transposases, but some TFs also have this domain, *ZBED6* being a typical example [74, 75]. Our study indicates that maize has many BED family members. This may be related to the abundance of transposable elements in the maize genome [19].

The CSD family has a cold-shocked domain (CSD) resembling the domain possessed by bacterial cold-shocked proteins, its first member was identified in cold-shock stimulation [76]. The CSD domains in plant CSD TFs still exhibit high similarity to bacterial ones [77]. The HMG and HMGI/HMGY families belong to the same big group of proteins, the High-mobility group [78]. HMG proteins possess the HMG-box domain, and they are involved in many processes related to DNA replication, transcription, and DNA repair. They also interact with other TFs [78]. HMGI/HMGY proteins possess the AT-hook domain, which binds to AT-rich DNA sequences [26, 79, 80]. In plants, some proteins in this family also contain a domain called DUF296 by Pfam (Pfam ID: PF03479, IntroPro ID: IPR005175), which confers the ability to interact with each other and essential for nuclear localization [81]. The MIZ family derived its name from the fact that one of its member in human, *MIZ1* (Msx-interacting-zinc finger), interacts with another transcription factor *MSX2* [82]. *MIZ1* is able to act as DNA-binding transcription factor and can increase the binding affinity of *MSX2* through their interaction.

### Comparison with PlantTFcat

We compared our annotation with that of PlantTFcat, which classified a collection of 108 families of TFs, transcriptional regulators (TRs), chromatin regulators (CRs) and some proteins that function as basal transcription machineries, according to combinations of InterPro domain annotations [15, 83]. In our annotation of maize TFs and TCs, we used the TF annotation of maize FGS in PlantTFDB 3.0 as the reference dataset, which contains 3316 TF proteins [17].

According to the website of PlantTFcat, there are 59 TF families in PlantTFcat that are related to 51 TF families in PlantTFDB 3.0. Our annotation contained 3151 proteins in these 51 families and recovered 3121 proteins annotated by PlantTFDB 3.0 (Additional file 10: Table S9 (A)). PlantTFcat predicted 6809 proteins related to TF, TR or CR, only 4888 of which are found in families defined by PlantTFDB 3.0 (48 families) and overlapped with 3121 proteins annotated by PlantTFDB 3.0 (Additional file 10: Table S9(B)). PlantTFcat predicted more TF proteins because they considered more domains in some families. For example, PlantTFcat also considered InterPro domain IPR001841 in the C2H2 family, in which the annotated domain is the RING type zinc finger domain. For classification, we classified 3119 proteins into the families annotated by PlantTFDB 3.0 (Additional file 10: Table S9 (A)), whereas the corresponding number for PlantTFcat is only 2767 (Additional file 10: Table S9 (B)); this difference might be due to the fact that the classification rules are not exactly the same for PlantTFcat and PlantTFDB 3.0.

We also considered the possibility that PlantTFcat may also include some TFs in the 7 families defined by PlantTFDB 3.0 that have no defined correspondence to PlantTFcat families. We conducted a test, using all 3316 TFs annotated by PlantTFDB 3.0 as the reference dataset. Our annotation recovered 3301 of them but the corresponding number for PlantTFcat was only 3267 (Additional file 10: Tables S9 (A) ~ (D)). Among the 15 TFs that are not in our annotation, seven are pseudogenes, five are transposons, two have FDs and one has a DBD with a score lower than our threshold (Additional file 10: Table S9 (D). Our annotation assigned 3280 TFs to correct families, whereas the corresponding number for PlantTFcat was only 2767 (Additional file 10: Tables S9 (A) and S9 (B)).

We also repeated the same set of tests using the millet Yugu1 TFs annotated by PlantTFDB 3.0 as the reference dataset. We first considered the 51 families in PlantTFDB 3.0 that correspond to 59 PlantTFcat families. Our annotation in Yugu1 covered 1909 proteins, which overlapped with 1896 proteins in the corresponding families in PlantTFDB 3.0 (Additional file 10: Table S9 (E)). PlantTFcat identified 4285 proteins that were TFs, TRs or CRs, 2942 of which belonged to 49 of the 51 families defined by PlantTFDB 3.0 and overlapped with 1891 proteins in these families in PlantTFDB 3.0 (Additional file 10: Table S9 (F)). Among the 1891 proteins recovered by PlantTFcat, only 1701 were assigned to correct families in PlantTFDB 3.0 (Additional file 10: Table S9 (F)), while in our annotation 1894 of the 1896 proteins were assigned to the correct families (Additional file 10: Table S9 (E)).

When using all 1994 Yugu1 TFs in PlantTFDB 3.0 as the reference set, our annotation recovered 1992 of them, while PlantTFcat only recovered 1966 (Additional file 10: Tables S9 (E) ~ (H)). The only two TFs we did not recover had FDs considered by our classification rules and so were not qualified to be TFs (Additional file 1: Table S1). When considering family assignment, we assigned 1986 TFs to the correct families (Additional file 10: Table S9 (E)), while PlantTFcat only assigned 1701 TFs to the correct families (Additional file 10: Table S9 (F)). Compared to PlantTFcat, our annotation has better coverage and higher accuracy in identifying and classifying Yugu1 TFs.

In summary, our recovery rate was slightly higher than that of PlantTFcat for the two benchmark datasets. Our method could correctly assign more maize and millet TFs into families in PlantTFDB 3.0 that were also shared by PlantTFcat.

### Preferential expression of TF and TC gene families in tissues

We assessed the importance of TF and TC families in some tissues by gene set enrichment analysis. Our analysis took advantage of the abundant RNA-seq data in maize and the RNA-seq data from the millet genome projects, so that we could cover different developmental stages of some tissues. We identified 39 TF families and 4 TC families in maize enriched in expression in one or more tissue groups (Table 5); the corresponding numbers in millet were 28 and 2, respectively (Table 6). There were 7 TF families and 2 TC families in maize enriched in all tissue groups, and the corresponding numbers in millets were 3 and 1, respectively (Tables 5 and 6). Our cross-comparison between these two species indicated that 19 TF families and 2 TC families were enriched in the same tissue group in both species, suggesting expression conservation of these TF and TC families (Tables 5 and 6). Prevalent expression of WRKY and NAC genes in root, NF-YA and NF-YC genes in leaf, and SBP genes in tassel are good examples (Tables 5 and 6). The similarity of enrichment pattern among different tissues may reflect the physiological similarity in those tissues.

### TF and TC genes preferentially expressed in certain tissues

Even though enrichment data may tell us in which tissue and under which conditions a TF or TC family may be functional, we still need to check the expression preference of individual TF or TC genes, because there may be cases in which only a small fraction of genes in certain TF or TC families exhibit expression preference in particular tissues or conditions, a situation that cannot be revealed by gene set enrichment analysis. We identified 1819 TF genes and 109 TC genes in maize preferentially expressed in at least one tissue (Additional file 3: Tables S3 (A) and S3 (B)). We also identified 608 TF genes and 33 TC genes in millet preferentially expressed in at least one tissue (Additional file 3: Tables S3 (E) and S3 (F)). Among those preferentially expressed TF and TC genes, we identified 138 TF and 7 TC maize-millet orthologous gene pairs with conserved differential expression (Additional file 3: Tables S3 (A), S3 (B), S3 (E) and S3 (F)). In terms of the number of preferentially expressed TF and TC genes in millet, a substantial proportion of them have conserved expression preference. Our result can be useful for unraveling specific biological process regulated by those TFs and TCs.

## Conclusion

We identified a set of TF and a set of TC families from current database annotation and experimental evidence and conducted a genome-wide prediction of TF and TC genes in maize and millet. We identified many TF and TC families that have TF or TC functions but have not been curated by known plant TF databases, and we studied the evolutionary relationships among the members of a new family. Our annotation quality is comparable to or better than those obtained by other approaches. We provided supporting evidence for our predictions from gene expression data in maize or millet and from orthologous genes in Arabidopsis or rice. We evaluated the expression preference of TF and TC genes in tissues in these two species and found a substantial proportion of these genes exhibiting conserved expression preference between the two species. We also identified C4-related TF and TC genes, using the published data from maize and millet BS and M cells, and also from the foliar leaves and husk leaves of maize. Our study significantly expanded current TF and TC annotations in maize and millet, facilitating the study of regulation of gene expression and tissue morphogenesis in maize and millet.

## Methods

### Genome annotation of maize and millet

Genome annotation and protein sequences of the maize (*Zea mays*) WGS were downloaded from maizesequence.org (http://ftp.maizesequence.org/release-5b/). Genome annotation and protein sequences of *Setaria italica* Yugu1 were downloaded from Ensembl Plants release 17 and those of *Setaria italica* Zhang gu were downloaded from Foxtail Millet Database (http://foxtailmillet.genomics.org.cn) [27].

### Prediction of protein domains

We collected TF and TC domains from PlantTFDB 3.0, Grassius, TreeTFDB, AnimalTFDB, ProFITS and PlnTFDB 3.0 [11, 13, 14, 16, 17, 23]. We also included Pfam domains that have GO annotations related to TC, or other supporting evidences such as ChIP-seq and PBM that suggest DNA binding capability of proteins having them [25, 26, 84]. The GO annotation of Pfam domains was inferred by GO annotation of their corresponding InterPro domains (Additional file 2: Table S2) [83].

We considered 3 types of protein domain: DNA-binding domain (DBD), auxiliary domain (AD), and forbidden domain (FD). A domain is a FD if its existence in a protein forbids it to have TF function, even if it contains a DBD. For example, a protein with a C2H2 domain and also an RNase_T domain, which is a FD, is not considered a TF. An AD is a protein domain that enables a TF to respond to a specific signal and TFs with the same DBD but with different ADs are usually classified into different TF families.

The protein domains are represented as characteristic motifs of protein segment by Hidden Markov Models (HMMs). Most of the HMM models were collected from Pfam 27.0 [84]. For those domains without any HMM model in Pfam 27.0, we downloaded the multiple sequence alignment of TF domains from PlantTFDB 2.0 and PlnTFDB 3.0 (Additional file 2: Table S2) and obtained corresponding HMM models by using the hmmbuild function in HMMER 3.0 [11, 85]. The presence of protein domains in protein sequences of maize, millet Yugu1 and Zhang gu was predicted by using the hmmsearch function in HMMER 3.0. Similar to Pfam and PlantTFDB 3.0, we used the bit scores output by HMMER 3.0 as a metric for deciding the thresholds for classifying domains. As in Pfam and PlantTFDB 3.0, there are two different measurements for each domain: domain cutoff and sequence cutoff. For each Pfam domain related to TF families in PlantTFDB 3.0, we compared the domain cutoff and sequence cutoff of PlantTFDB 2.0 and the noise cutoff of Pfam, and we selected the minimum value as the corresponding domain cutoff and sequence cutoff (Additional file 2: Table S2). For each of the other domains covered by Pfam 27.0, we used the noise cutoff of domain cutoff and sequence cutoff suggested by Pfam 27.0 (Additional file 2: Table S2). For each of G2-like, NF-YB, NF-YC and Trihelix families, we used the hmmsearch function in HMMER 3.0 to obtain bit scores of DBDs in maize and millet TFs in PlantTFDB 3.0, and we selected the maize or millet TF in the family that with the lowest domain cutoff score and sequence cutoff score and used these two scores as the threshold (Additional file 2: Table S2). For thresholds of HRT-like, SAP, STAT and VOZ families, we used the thresholds suggested by PlantTFDB 2.0 (Additional file 2: Table S2).

### Family assignment rules

As in PlantTFDB 3.0, our TF classification considers the DBD, AD and FD domains (Additional file 2: Table S2) [17]. Our assignment rules are briefly as follows:

1. If a protein sequence has one or more DBDs and it has no AD and FD, we assign it to a TF family according to its DBD.
2. If a protein sequence contains one or more DBD and Ads, but no FD, we assign it to the family that contains the specific DBD and ADs.

Let us use AtHB8 as an example. First, since AtHB8 has a homeobox domain but no FD, it belongs to the homeobox superfamily. Second, since it has a START domain, it is classified into the HD-ZIP family, because START is the AD required for the HD-ZIP family (Additional file 1: Table S1).

Our TC classification procedure is the same as above, except that we now consider the TC domain instead of the DBD domain (Additional file 1: Table S1).

### Performance comparison

We compared our annotation with that of PlantTFcat [15]. The annotations of maize and millet Yugu1 TFs on PlantTFDB 3.0 were used as two independent benchmark datasets [17]. We conducted another TF prediction by using PlantTFcat on maize FGS and current millet Yugu1 annotation. We compared the performance of our pipeline and PlantTFcat in two ways. The first one is to evaluate coverage and classification accuracy on the 51 PlantTFDB families that have correspondences in PlantTFcat families, which were defined on PlantTFcat website (http://plantgrn.noble.org/PlantTFcat/). Second, we compared the coverage on the whole benchmark set. The coverage was defined according to the overlap between the prediction result and benchmark datasets. The classification accuracy was defined as the proportion of proteins that could be assigned to correct families in the benchmark datasets.

### Orthologs of TF and TC genes in maize and millet

For TF and TC genes in maize and millet Yugu1, the orthologous relationships we used were obtained from Ensembl Plants release 17 [27]. For each TF or TC protein we predicted, we used BLASTP to find its best ortholog in other species, i.e., the E-value of the best high-scoring segment pair (HSP) should be smaller than 1e-20 and the length of subsequence included in the HSP in both sequences must occupy at least 30% of the total length in both sequences. The same procedure was applied to millet Zhang gu TF and TC genes.

### Expression analysis of RNA-Seq and microarray datasets

For TF and TC genes in maize, we collected 8 RNA-seq datasets (Additional file 4: Table S4) and the microarray dataset from Sekhon *et al*. [20, 28–35]. Raw FPKM (Fragments Per Kilobase of transcript per Million mapped reads) values of RNA-seq datasets except Liu *et al*. were downloaded from qTeller (http://qteller.com) [20]. For the RNA-seq datasets in maize and millet, we normalized the FPKM values of genes by the quantile normalization method [86]. We regarded a gene as expressed if it satisfied one of the following conditions:

1. For the RNA-seq datasets, the gene must have FPKM ≥ 1 in at least one sample (Additional file 5: Table S5).
2. For the microarray dataset, the gene must have log2-transformed expression value ≥ 7.65 in at least one condition.

For condition 2, we downloaded the dataset corresponding to maize genome release 5a.59 from PLEXdb (PLEXdb accession No. ZM37) [87].

We separated the samples in RNA-seq datasets into 7 tissue groups: seed, mature leaf, embryonic leaf, root, shoot, ear and tassel. We used this grouping for enrichment analysis and identification of preferentially expressed TF and TC genes. Additional file 4: Table S4 shows the grouping of RNA-seq samples.

For detecting differential expression in maize genes, we transformed the normalized FPKM values to z-scores. If a gene has a z-score ≧3 in a tissue, this gene is defined as preferentially expressed in this tissue.

For millet Zhang gu, we analyzed the expression data of 4 different tissues downloaded from the Foxtail Millet Database. We regarded a gene as expressed if it had FPKM ≥ 1 in at least one sample (Additional file 6: Table S6).

For the identification of preferentially expressed TF and TC genes in millet, since only four different tissues were studied, we did not apply z-transformation. We say that a gene is preferentially expressed in a tissue if the normalized FPKM value in that tissue is two times larger than the normalized FPKM values in the others tissues.

To identify TF and TC genes with preferential expression conserved in maize and millet Zhang gu, we considered reciprocal best hit pairs according to result of BLASTP. For a pair of maize and millet genes, if they are both preferentially expressed in a tissue, we say they have conserved differential expression in that tissue. For maize, embryonic leaf and leaf at later stage are grouped together as “Leaf”. If a millet gene preferentially expressed in mature leaf and its counterpart in maize preferentially expressed in either mature leaf or embryonic leaf, it has conserved differential expression.

### Enrichment analysis

For maize, we grouped the RNA-seq samples into 7 tissue groups (Additional file 4: Table S4) and examined whether there were TF or TC families significantly preferentially expressed in certain groups. For millet Zhang gu, we treated 4 samples as 4 groups (Additional file 4: Table S4). In both cultivars, all TF and TC families with more than 5 genes were used in our analysis. Fisher’s exact test was used to check statistical significance (i.e., p-value < 0.05).

### Phylogenetic analysis

We considered the 9 TF families not included by PlantTFDB 3.0 (Table 1). The domain annotation and protein sequences of Physcomitrella patens v3.0 was downloaded from Phytozome 9.1 (Physcomitrella patens v3.0 early release, DOE-JGI, http://www.phytozome.net/physcomitrella_er.php), the annotation of the other species we considered was downloaded from Ensembl via Ensembl Biomart [27, 88, 89]. The members in the 9 TF families in other species were identified based on the existence of DBDs in these families. Detailed information about genome annotation and protein sequences we used are described in Additional file 9.

For reconstructing the phylogenetic tree of the members of a gene family, the protein sequences in the family were first aligned using MUSCLE [90]. A phylogenetic tree was then constructed by the Neighbor-Joining (NJ) method with the bootstrap procedure repeated 1 000 times, using MEGA5 [91].

## Electronic supplementary material

Additional file 1: Table S1: Classification rules for TF and TC families. For each family, we indicated whether it is TF or TC, the corresponding family in PlantTFDB 3.0, related families in PlantTFcat, required domain(s), auxiliary domain(s) (if any), forbidden domain(s) (if any), referenced sources (TF databases, Gene Ontology website, literature, etc. ), and superfamily it belongs according to PlantTFDB 3.0 (if any). (XLS 50 KB)

Additional file 2: Table S2: Protein domains used in our studies and corresponding thresholds we used in domain prediction. For each domain, we indicate its type (DBD, AD, FD, TC domain), source of the domain (Pfam or self-built, see Methods), Pfam ID (if any), InterPro ID (if any), sequence cutoff and domain cutoff (in bit score). (XLS 68 KB)

Additional file 3: Table S3: TF and TC genes classified in maize and millet. For each TF/TC gene, we indicated their expression preference (tissue group it expressed/preferentially expressed, and whether its orthologs in maize or millet Zhang gu also preferentially expressed in same tissue group). We also listed all TFs/TCs encoded by each TF/TC genes, families of these TFs/TCs, and best BLASTP hit of these TFs/TCs in other genomes. For maize TF genes and TC genes, we also indicated whether they have support from microarray data from Sekhon *et al*. [31], and whether they are in FGS. (A) Annotation of maize TF genes. (B) Annotation of maize TC genes. (C) Annotation of millet Yugu1 TF genes. (D) Annotation of millet Yugu1 TC genes. (E) Annotation of millet Zhang gu TF genes. (F) Annotation of millet TC genes. (XLS 2 MB)

Additional file 4: Table S4: RNA-seq samples used in our study. For each sample, we listed tissue group it belongs to, and also the reference of the sample. (XLS 30 KB)

Additional file 5: Table S5: Normalized expression values of all maize protein-coding genes in all RNA-seq samples. (XLSX 16 MB)

Additional file 6: Table S6: Normalized expression values of all millet Zhang gu genes in all RNA-seq samples. (XLSX 2 MB)

Additional file 7: Table S7: C4-related TF and TC genes in maize and millet. (A) C4-related maize TF genes annotated from previous studies. (B) C4-related maize TC genes annotated from previous studies (C) C4-related millet TF genes annotated from previous studies (D) C4-related millet TC genes annotated from previous studies. Each gene is annotated with its expression preference identified in previous studies. (BS: bundle sheath cells, BS_con: the differential expression preference in BS cells conserved in S. viridis and maize, M: mesophyll cells, M_con: the differential expression preference in M cells conserved in maize and millet, positive kranz: possible positive regulator for kranz anatomy, negative kranz: possible negative kranz regulator for kranz anatomy). (XLSX 66 KB)

Additional file 8: Figure S1-S9: Phylogenetic trees of 9 newly annotated families in various species. Sequences used in constructing these phylogenetic trees and version of genome annotation information are described in Table S9. The method for constructing phylogenetic trees is described in Methods. **Figure S1.** Phylogenetic tree of Pseudo ARR-B family. **Figure S2.** Phylogenetic tree of mTERF family. **Figure S3.** Phylogenetic tree of MBD family. **Figure S4.** Phylogenetic tree of LITAF family. **Figure S5.** Phylogenetic tree of BED family. **Figure S6.** Phylogenetic tree of CSD family. **Figure S7.** Phylogenetic tree of HMG family. **Figure S8.** Phylogenetic tree of HMGI/HMGY family. **Figure S9.** Phylogenetic tree of MIZ family. (PDF 75 KB)

Additional file 9: Table S8: Annotation of amino acid sequences used in constructing phylogenetic trees of 9 newly annotated TF families in species we considered, and the information of genome annotation of these species. (A) Genome annotation information of species considered in construction of phylogenetic trees. (B) Information of sequences used in constructing the phylogenetic tree of BED family. (C) Information of sequences used in constructing the phylogenetic tree of CSD family. (D) Information of sequences used in constructing the phylogenetic tree of HMG family. (E) Information of sequences used in constructing the phylogenetic tree of HMGI/HMGY family. (F) Information of sequences used in constructing the phylogenetic tree of LITAF family. (G) Information of sequences used in constructing the phylogenetic tree of LITAF family. (H) Information of sequences used in constructing the phylogenetic tree of MBD family. (I) Information of sequences used in constructing the phylogenetic tree of MIZ family. (J) Information of sequences used in constructing the phylogenetic tree of Pseudo ARR-B family. (XLSX 59 KB)

Additional file 10: Table S9: Performance evaluation of our annotation. We used TF annotation of maize FGS and millet Yugu1 on PlantTFDB 3.0 as two independent benchmark datasets to assess the quality of our annotation and compare it with PlantTFcat. (A) Comparison between our annotation and the PlantTFDB 3.0 annotation of maize FGS TFs. (B) Comparison between annotation of PlantTFcat and the PlantTFDB 3.0 annotation of maize FGS TFs. (C) Maize TFs annotated in PlantTFDB 3.0 but not covered by our annotation. (D) Maize TFs annotated in PlantTFDB 3.0 but not covered by PlantTFcat. (E) Comparison between our annotation and the PlantTFDB 3.0 annotation of millet Yugu1 TFs. Table (F) Comparison between annotation of PlantTFcat and the PlantTFDB 3.0 annotation of millet Yugu1 TFs. (G) Millet Yugu1 TFs annotated by PlantTFDB 3.0 but not covered by our annotation. (H) Millet Yugu1 TFs annotated by PlantTFDB 3.0 but not covered by PlantTFcat. (XLS 2 MB)
