# Supplementary figures and images for: Maize and millet transcription factors annotated using comparative genomic and transcriptomic data
Source: BMC Genomics. 2014 Sep 27;15(1):818. doi: 10.1186/1471-2164-15-818 (PMC4189582; doi:10.1186/1471-2164-15-818)

Fig. S1

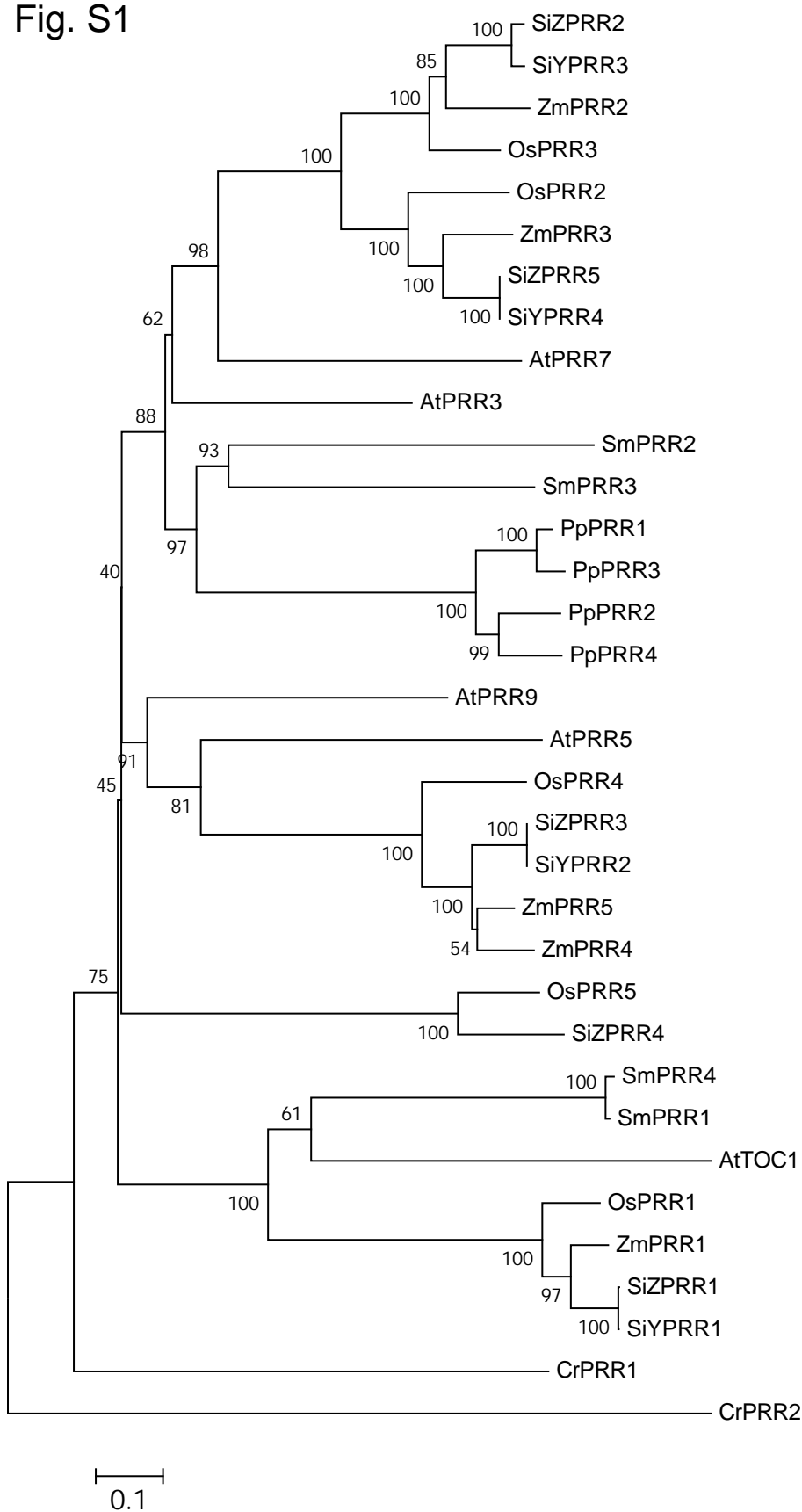

**Fig. S2**

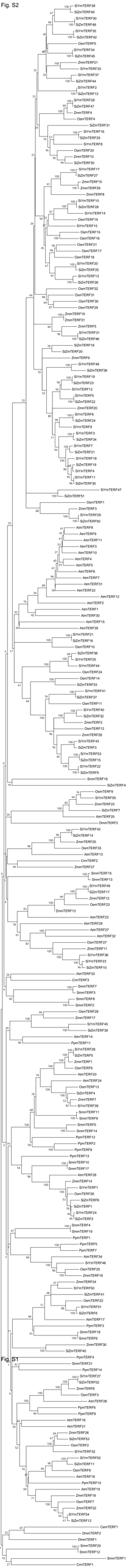

**Fig. S1**

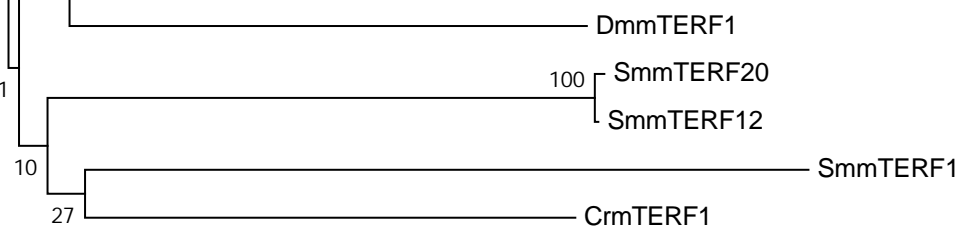

Fig. S3

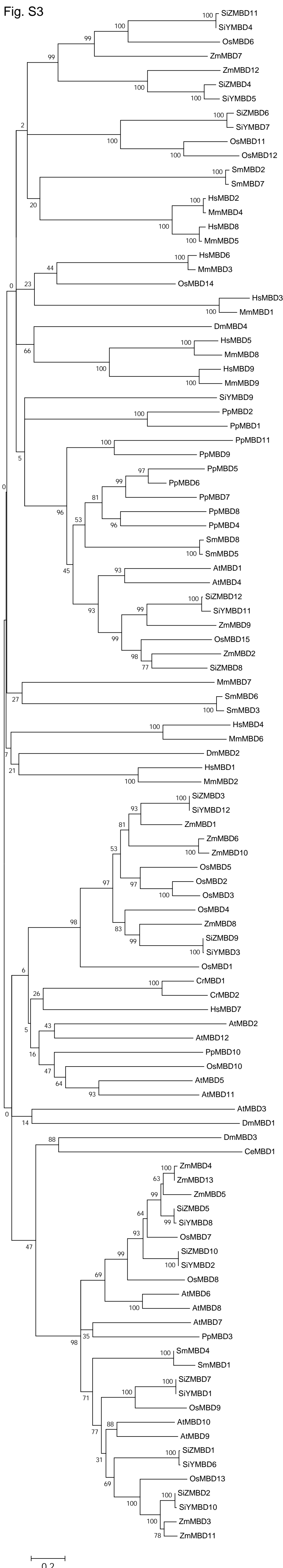

Fig. S4

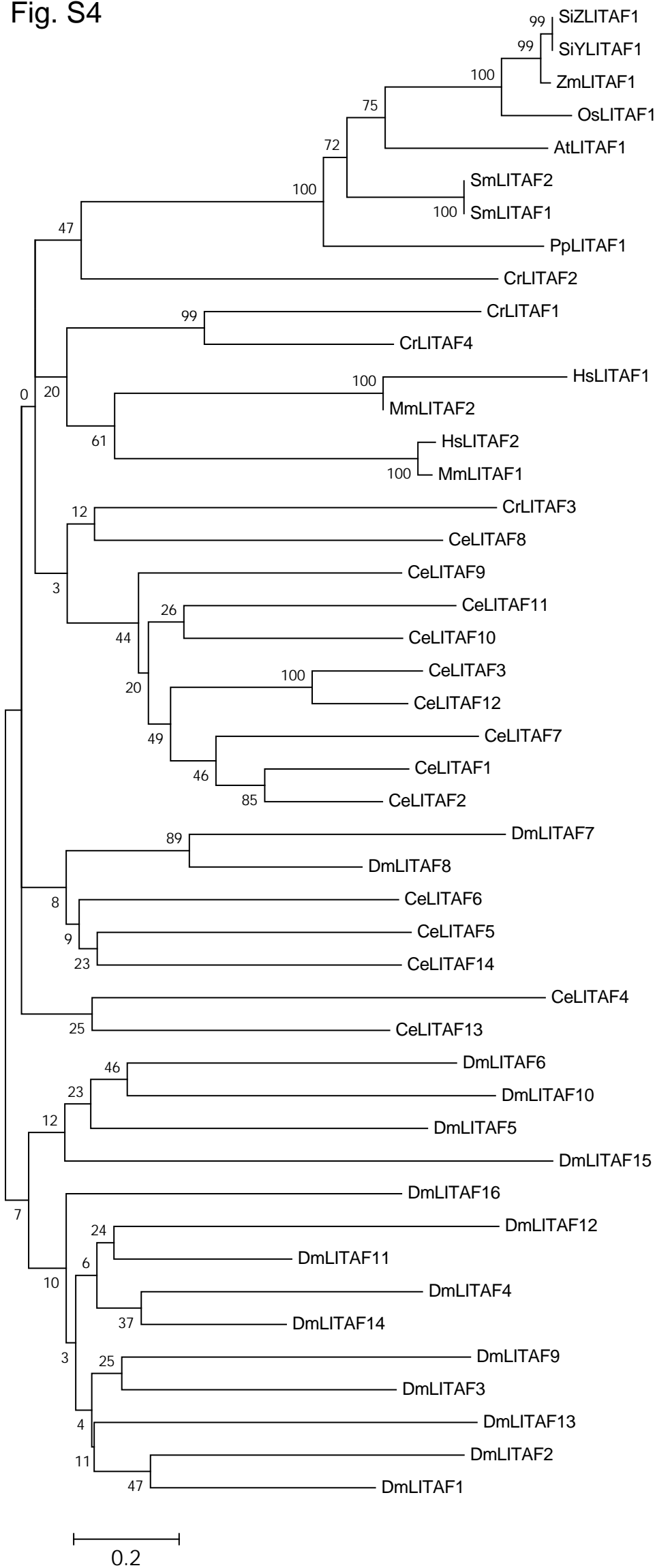

Fig. S5

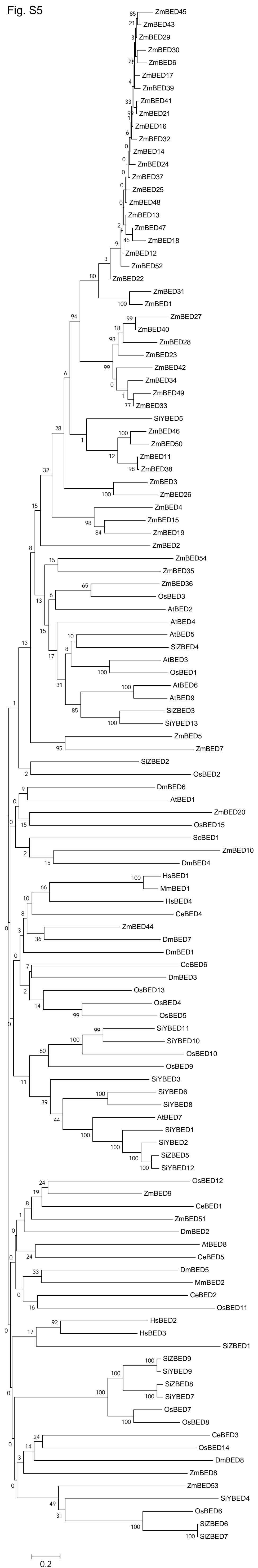

Fig. S6

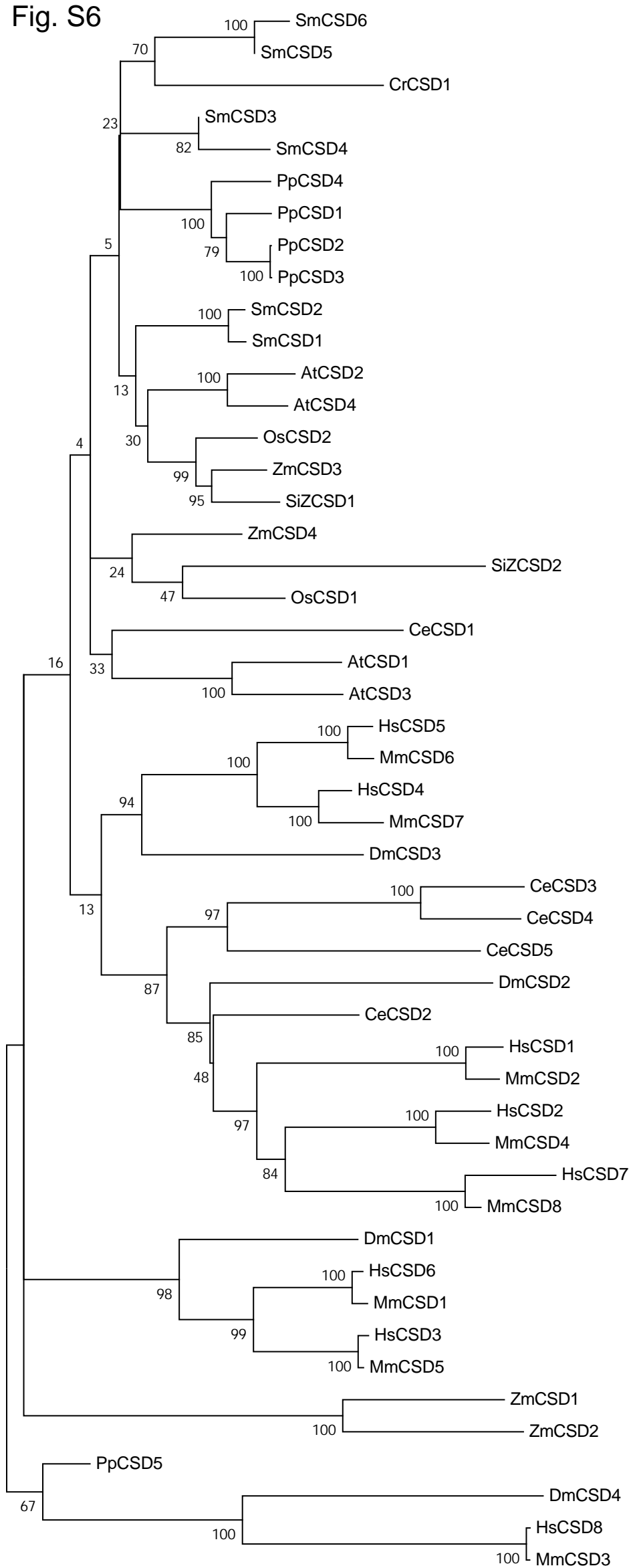

0.1

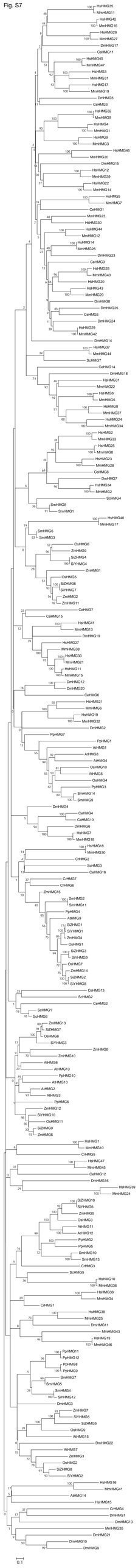

Fig. S8

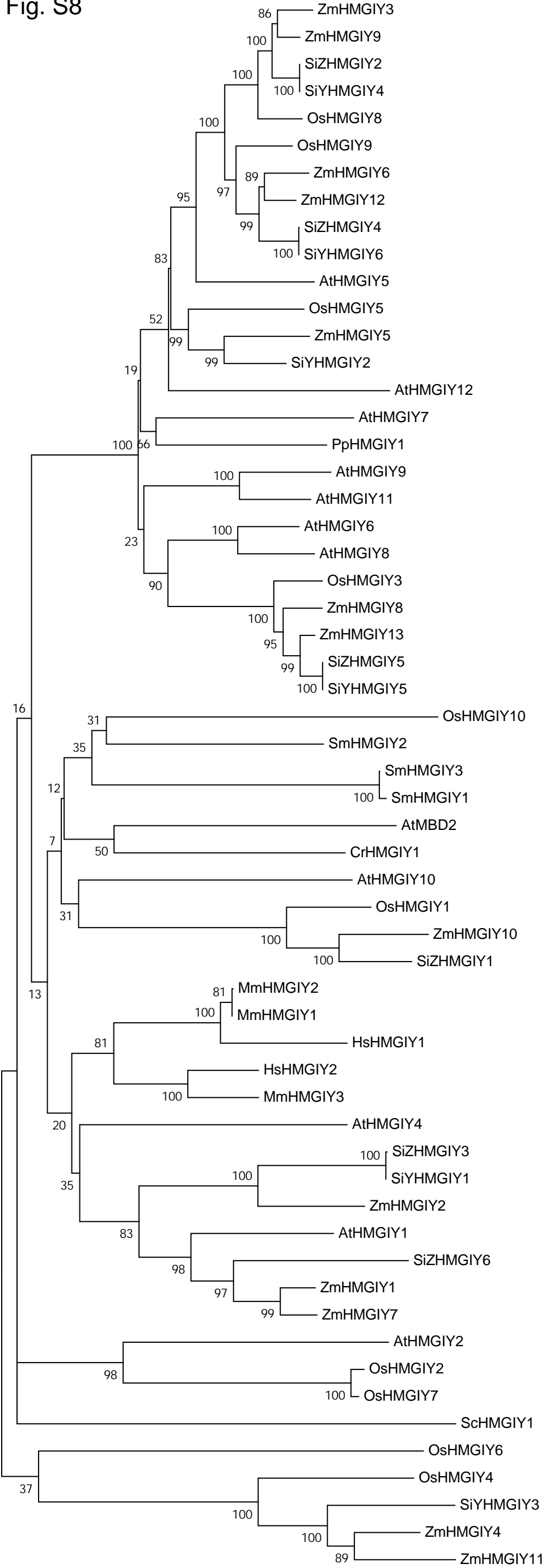

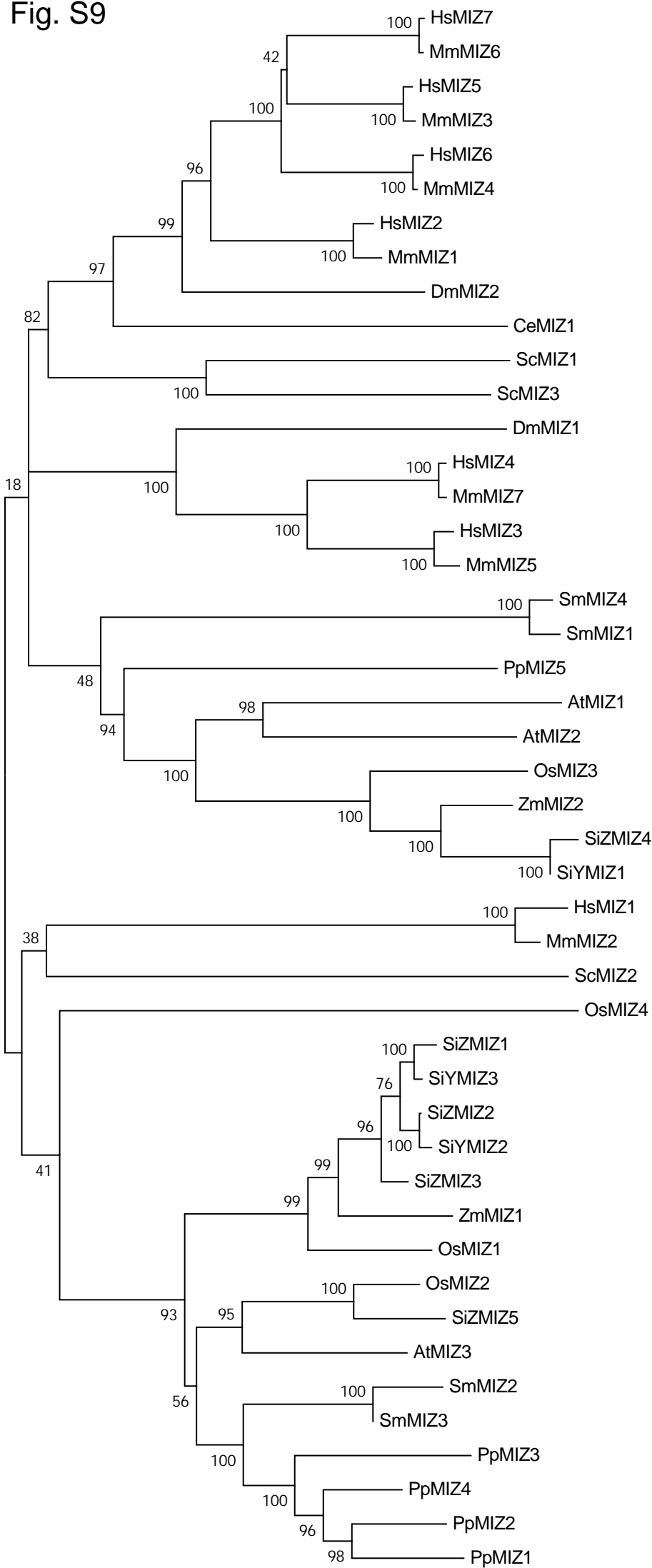

Supplement: Supplementary file 8 — Additional file 8: Figure S1-S9: Phylogenetic trees of 9 newly annotated families in various species. Sequences used in constructing these phylogenetic trees and version of genome annotation information are described in Table S9. The method for constructing phylogenetic trees is described in Methods. Figure S1. Phylogenetic tree of Pseudo ARR-B family. Figure S2. Phylogenetic tree of mTERF family. Figure S3. Phylogenetic tree of MBD family. Figure S4. Phylogenetic tree of LITAF family. Figure S5. Phylogenetic tree of BED family. Figure S6. Phylogenetic tree of CSD family. Figure S7. Phylogenetic tree of HMG family. Figure S8. Phylogenetic tree of HMGI/HMGY family. Figure S9. Phylogenetic tree of MIZ family. (PDF 75 KB) [file 12864_2014_6495_MOESM8_ESM.pdf]
